# A trypanosome trifecta: an independently tunable triple inducible system for genetic studies in *Trypanosoma brucei*

**DOI:** 10.1128/msphere.00596-25

**Published:** 2026-03-26

**Authors:** Matt J. Romprey, Raveen Armstrong, Peter McGill, Cara M. Jenkins, Justin H. Li, Michele M. Klingbeil

**Affiliations:** 1 Department of Microbiology, University of Massachusetts 196202, Amherst, Massachusetts, USA; 2 Institute for Applied Life Sciences, University of Massachusetts 465576, Amherst, Massachusetts, USA; California State University Fullerton, Fullerton, California, USA

**Keywords:** *Trypanosoma*, cumate, inducible expression, kinetoplast, vanillic acid, tetracycline, RNAi complementation, maxicircle, DNA polymerase

## Abstract

*Trypanosoma brucei* is a model unicellular parasite for cellular and molecular genetic studies, but tools for more multiplexed experiments are limited. Tetracycline (Tet)-inducible gene regulation has been a long-standing and effective approach for overexpression, RNAi, and genome-wide screens and has been foundational for studying essential genes that are required for biological processes and identifying potential drug targets. To achieve greater flexibility in experimental design, we capitalized upon previously described dual inducer systems that combined vanillic acid (Van) or cumate (Cym) with Tet as inducers. Here, we report the development of a triple inducible system combining Cym, Van, and Tet repressors to selectively regulate the expression of three genes within a single cell line called PHITER. To demonstrate independent control, we adapted the previously characterized Van/Tet dual inducible RNAi complementation cell line, IBComp^VaT^, for additional Cym inducible expression of an eGFP reporter. We provide evidence that each inducer operates independently with no evidence of leaky expression. In this system, Cym induction is specific and tunable. As proof of principle, we used triple induction for *POLIB* RNAi, complementation with a recoded ectopic POLIB-PTP variant, and overexpression of the maxicircle helicase. We confirmed that Cym inducible PIF2 overexpression resulted in massive overreplication of maxicircles that is suppressed when *POLIB* is silenced, thereby demonstrating a requirement for POLIB during replication stress. The triple inducible system enables the study of complex and pleiotropic phenotypes through a more sophisticated experimental design that could not previously be achieved and can be applied for the development of multiplexed genomic screens.

**IMPORTANCE:**

*Trypanosoma brucei* is a protist parasite that causes significant health and economic burden for sub-Saharan Africa and serves as a key model organism for a group of eukaryotes called the kinetoplastids. *T. brucei* and related parasites contain an interlocked mtDNA network called kinetoplast DNA (kDNA), composed of maxicircles and minicircles. Although *T. brucei* is highly genetically tractable, limitations exist for more complex experimental designs and multiplexed forward genetic screens. Here, we describe the first triple inducible system in *T. brucei* or any eukaryote that combines three independent repressors (cumate [Cym], tetracycline [Tet], and vanillic acid [Van]) to selectively regulate three genes within a single cell line. We demonstrate the utility of the system using an eGFP reporter and robust RNAi complementation of kDNA polymerase POLIB. Furthermore, we investigate the role of POLIB in maxicircle replication by overexpressing the maxicircle helicase during *POLIB* RNAi, revealing an early requirement for POLIB during replication stress.

## INTRODUCTION

The African trypanosome, *Trypanosoma brucei*, is a tsetse fly-transmitted parasitic protist of the supergroup Discoba that causes the neglected tropical disease human African trypanosomiasis and a related wasting disease in cattle called nagana (1, 2). In addition to medical and economic importance, *T. brucei* has served as a tractable model organism with many advanced genetics tools to uncover details about fundamental biological processes based on their distinctive biology. Examples include extensive post-transcriptional gene regulation, mitochondrial RNA editing which overturned years of molecular central dogma and unusual metabolic adaptations (3, 4). Another example of extreme biology is their mitochondrial DNA network called kinetoplast DNA (kDNA). Trypanosomes have one of the most topologically complex mitochondrial genomes in nature composed of maxicircles and minicircles that are catenated into a single network.

The kDNA structure and replication mechanism are divergent from all other eukaryotes and are essential for parasite survival and life cycle completion (5). A hallmark of kDNA replication is the topoisomerase II-mediated minicircle release and attachment mechanism, resulting in spatial and temporal separation of replication events, while maxicircles replicate still catenated within the network (6–8). Both mini and maxicircles undergo unidirectional theta structure replication, producing progeny that still contain a nick or a 1–2 nucleotide gap (9, 10). During later stages of replication, nicks and gaps are repaired before progeny networks undergo topological remodeling and finally segregate into daughter cells. Remarkably, minicircle progeny are attached to the network while still containing at least one gap. This “mark” on replicated progeny is a possible mechanism to ensure each circle is replicated just once per cell cycle. However, persistent gaps (ssDNA regions) negatively impact genome stability and can lead to replication stress, suggesting that the DNA damage tolerance response during kDNA replication is essential and therefore notably different in trypanosomes (9, 11).

The complexity of kDNA replication is also reflected in the many additional factors not present in mammalian nucleoids, including six DNA polymerases (Pol), six helicases, three topoisomerases, two primases, two ligases, and two origin binding proteins, among others. Greater than 50 proteins have roles in the replication and segregation of the DNA network, as well as maintaining the kDNA structural integrity, and have been summarized elsewhere (7, 8, 12, 13). Interestingly, many of the kDNA replication factors have non-redundant roles discovered using RNAi for reverse genetic analysis of gene function. For example, *T. brucei* uses three family A DNA Pol paralogs (POLIB, POLIC, POLID) that are independently essential; when one paralog is silenced, the others cannot compensate for the loss (14–18). Our central hypothesis is that divergent features of the DNA Pol paralogs facilitate specialized roles during kDNA replication. However, the precise division of labor among these Pol paralogs remains unanswered.

Tetracycline (Tet) inducible systems have been crucial for single gene and genome-wide RNAi studies over the past two decades for both insect procyclic form and mammalian bloodstream form parasites (19). Among the many improvements to trypanosome molecular biology constructs, independent dual inducible systems were developed using Tet and vanillic acid (Van) or Tet and cumate (Cym) (20, 21). We recently reported the use of a dual inducible system for independently regulating RNAi for *POLIB* knockdown and overexpression of an ectopic copy of an RNAi-resistant variant of POLIB through the addition of Van and Tet, respectively. This simultaneous induction allowed for robust RNAi complementation (add back experiment) to confirm the RNAi phenotype could be attributed to POLIB alone (22). RNAi complementation studies and their associated control experiments can now be performed within a single cell line.

This highly tractable system could be expanded for further experimental flexibility by adding a third inducer, thus allowing for the independent regulation of three processes in one cell line. In this triple inducible system, RNAi complementation could still operate using the Van and Tet repressors to concurrently control RNAi and overexpression, while the Cym repressor would allow for tunable regulation of an additional gene. Here, we describe the establishment of a triple inducer parental system that expresses three repressors and demonstrates independent and tunable Cym inducible expression using an eGFP reporter. To evaluate the full potential of a triple inducible system, we built upon the previously characterized Van/Tet dual inducible cell line for *POLIB* RNAi complementation (22) as proof of principle to demonstrate that three inductions can function independently and simultaneously. Importantly, we applied the triple inducible system to evaluate the role of POLIB in maxicircle replication using PIF2 overexpression as an elegant reporter during *POLIB* RNAi and complementation. This tool overcomes key limitations and opens new avenues for trypanosomatid functional genomics.

## MATERIALS AND METHODS

For primer sequences, refer to Table S1.For cell lines and associated modifications used in this study, refer to Table S2.

### DNA constructs

#### Single marker cumate inducible system

The hygromycin resistance gene was PCR amplified from pKOHyg (23) using primers UM147 and UM149 for subsequent Gibson assembly (NEB) with EcoRI and NcoI digested pSmOxNUS (21) to create pSmOxNUSHyg. The cumate repressor (CymR) gene and the hygromycin resistance gene (Hph) were then PCR amplified from pSmOxNUSHyg using primers UM159 and UM160 for Gibson assembly with StuI and BsiWI digested pDEX-CuO (21) to create pCuRO-eGFP.

#### Cumate repressor plasmid

The CymR and Hph genes were PCR amplified from pSmOxNUSHyg using primers UM192 and UM193 for subsequent Gibson assembly with NheI and SpeI digested pJ1173 (20) to create pCymRHyg, thus removing the T7 RNA polymerase, tetracycline repressor (TetR), and a puromycin resistance gene from pJ1173.

#### Cumate inducible expression vectors

The neomycin resistance gene was PCR amplified from pC-PTP-NEO (24) using primers UM184 and UM185 for subsequent Gibson assembly (NEB) with BsiWI and StuI digested pDEX-CuO (21) to create pCuO-eGFP. The Su9-DarT(E160A) sequence was excised from pCuONeo-mtDarT(E160A)flag using EcoRV and PacI. Full-length PIF2 was PCR amplified from *T. brucei* 427 gDNA using primers UM194 and UM195 for subsequent Gibson assembly to create a C-terminal in-frame fusion with a 3X flag tag called pCuO-PIF2FLAG.

### Trypanosome cell culture and transfection

The *Trypanosoma brucei brucei* procyclic cell line SMUMA (single marker UMass) contains the integrated plasmid pJ1173 (20) carrying T7 RNA polymerase (T7RNAP), tetracycline repressor (TetR), and vanillic acid repressor (VanR). SMUMA cells were cultured at 27°C in SDM-79 medium supplemented with 15% heat-inactivated fetal bovine serum and 1 µg/mL puromycin (Puro) (22). The IBComp^VaT^ cell line (22) was cultured in SDM-79 medium supplemented with blasticidin (10 μg/mL), phleomycin (2.5 μg/mL), and puromycin (1 μg/mL).

To create the Cym triple inducible reporter cell line, CuRO-eGFP, NotI linearized pCuRO-eGFP was transfected into SMUMA cells via nucleofection to integrate into the 177 bp locus and selected with 50 μg/mL hygromycin (Hyg). To create the triple inducible PHITER parental cell line that expresses three repressors (CymR, TetR, and VanR), SMUMA (22) was transfected with HindIII digested pCymRHyg via nucleofection to integrate into the β/α tubulin array and selected with 50 μg/mL Hyg. Verification that all three repressors are expressed in PHITER clones is provided in Fig. S1. Four clonal cell lines were chosen based on doubling times and subsequently transfected via nucleofection with NotI linearized pCuO-eGFP for integration into the 177 bp repeat region and selected with 50 µg/mL G418 to screen for Cym inducible protein expression. These cell lines are called eGFP^PHITER^ and were not further dilution cloned. All other transfected cell lines were additionally supplemented with the appropriate selectable drug prior to limiting dilution to obtain clonal cell lines.

The POLIB dual inducible cell line IBComp^VaT^ clone P5D1 (22) was transfected with HindIII digested pCymRHyg and selected with 50 μg/mL Hyg, resulting in IBComp^PHIT^ (Puro, Hyg, inducible triad). Clone P1D8 was chosen for this study based on doubling time and was subsequently transfected with NotI linearized pCuO-eGFP and selected with 50 µg/mL G418 to create the IBComp-eGFP^PHIT^ reporter cell line. Repressor expression was verified by northern blot and clone P1F12 was chosen for this study. IBComp^PHIT^ clone P1D8 was also transfected with NotI linearized pCuO-PIF2FLAG and selected with 50 µg/mL G418 to create the IBComp-PIF2^PHIT^ cell line. Following dilution cloning, clone P1D4 was chosen based on the expression of PIF2-FLAG protein for further characterization.

### Inducible expression

Expression of eGFP was induced with 25 μg/mL of Cym for 48 h for both triple inducible reporter cell lines CuRO-eGFP and eGFP^PHITER^. In the proof-of-concept cell line, IBComp-eGFP^PHIT^, RNAi was induced by the addition of Van (250 µM dissolved in DMSO), and expression of an ectopic recoded version of wild-type POLIB PTP-tagged variant (POLIB_rec_-PTP) was induced by the addition of Tet (4 µg/mL). For the triple inducible experiment with PIF2-FLAG, RNAi was induced by the addition of 50 µM Van (in DMSO). Cultures were supplemented daily with Van and/or Tet to maintain RNAi and protein expression (22). For eGFP expression, cells were induced with Cym (3 μg/mL dissolved in DMSO) on transfer days. To assess if Cym induction was reversible, IBComp-eGFP^PHIT^ cells were grown for 2 days in the presence of 3 μg/mL Cym only or in the presence of all three inducers. Cells were then pelleted and washed once in 1× phosphate-buffered saline (PBS) to remove the inducer. Cells were resuspended and grown in media lacking Cym for an additional 3 days. Cells were then harvested at the indicated time points for SDS-PAGE and western blot analyses. For PIF2-FLAG expression, cells were induced with Cym (25 μg/mL dissolved in DMSO) supplemented daily.

### SDS-PAGE and western blotting

Cells were pelleted, washed with 1× PBS, supplemented with 1× protease inhibitor cocktail (Roche), resuspended, and stored at −80°C. Protein samples were fractionated in a sodium-dodecyl-sulfate polyacrylamide gel (SDS-PAGE) (150 V) and transferred overnight (90 mA) onto a PVDF membrane. Membranes were then blocked in a 5% wt/vol non-fat dry milk solution for 2 h.

PTP-tagged POLIB was detected with peroxidase anti-peroxidase soluble complex (PAP, 1:2,000, Sigma). eGFP was detected using anti-GFP rabbit polyclonal antibody (1:1,000, 1 h, Abcam), followed by HRP-conjugated goat anti-rabbit polyclonal antibody (1:5,000, 1 h, Pierce). TetR was detected using anti-TetR mouse monoclonal antibody (1:1,000, 1 h, Takara). Loading control was detected with mouse monoclonal anti-EF1α1 (1:15,000, 1 h, Santa Cruz Biotechnologies) and HRP-conjugated goat anti-mouse polyclonal antibody (1:2,500, 1 h, Sigma). 3×-FLAG-tagged PIF2 was detected using DYKDDDDK tag (D6W5B) rabbit monoclonal antibody (1:500, 1 h, Cell Signaling Technology), followed by HRP-conjugated goat anti-rabbit polyclonal antibody (1:5,000, 1 h, Pierce). Membranes were washed thrice with 1× Tris-buffered saline + Tween 20 after each antibody incubation. Chemiluminescent substrate (SuperSignal West Pico Plus, ThermoFisher) was used to detect protein. Band intensities were quantified using ImageJ software (http://imagej.nih.gov/ij/).

### RNA isolation and northern analysis

A total of 5 × 10^7^ cell equivalents were pelleted and washed with PBS for total RNA isolation with TRIreagent (Sigma-Aldrich). RNA was run on a 1.5% agarose/7% formaldehyde gel overnight at 25 V, then transferred to a Pall 60208 Biodyne B Membrane as previously described (22). RNA was cross-linked at 1,200 J/cm^2^ using a UV Stratalinker 1800 (Stratagene). Specific ^32^P-labeled probes were generated as described previously (15, 18) using PCR products for CymR, TetR, VanR, and POLIB (Table S1) using the Random Primers DNA labeling system (Invitrogen). Tubulin probes were generated from a 650 bp gel-extracted product from pZJMα (25). Hybridization and wash conditions were described previously (18). Specific mRNAs were detected and quantified using a Typhoon 9500 Phosphorimager (GE Healthcare) and normalized to the tubulin signal.

### DNA isolation and Southern blot analysis

Total DNA was isolated from 1 × 10^8^ cells using the Puregene Core Kit A (Qiagen) as previously described (18, 22). Fold change of maxicircle content was confirmed by dot-blot Southern hybridization using the following method: DNA from 1 × 10^6^ cells was treated with 0.2 N HCl for 15 min, neutralized to 165 mM Tris (pH 7.5)/82.5 mM NaCl before adding an equal volume of 2× denaturing solution (500 mM NaOH/20 mM EDTA, pH 8.0). Samples were incubated at 95°C for 10 min and then placed on ice for 2 min. Samples were then adjusted to 10× SSC. Biodyne B Membrane (Pall Corporation) was briefly washed with H_2_O and then equilibrated with 10× SSC. Membrane was placed on a 96-well BioDot SF Microfiltration Apparatus (Bio-Rad). After applying and washing samples according to the manufacturer’s directions, blots were UV cross-linked at 1,200 J/cm^2^. Maxicircles were detected with a randomly primed ^32^P-labeled PCR product (see Table S1 for primers). Hybridization and wash conditions were described as above. Images were acquired using a Typhoon 9500 Phosphorimager. All signals were quantified using ImageJ with background subtraction in duplicate and averaged, then normalized against the tubulin signal.

### IF microscopy

Cells were harvested at 1,000 × *g*, washed once, and resuspended in 1× PBS, then adhered to poly-L-lysine-coated slides (5 min). Cells were fixed with 3% paraformaldehyde (5 min), washed twice with 1× PBS + 0.1 M glycine, and then permeabilized with 0.05% Triton X-100 (5 min). Cells were washed thrice with 1× PBS. POLIB_rec_-PTP was detected with rabbit polyclonal anti-protein A (1:1,000, 1 h, Sigma), followed by secondary antibody Alexa Fluor 594 goat anti-rabbit (1:250, 1 h, ThermoFisher). eGFP was detected using rabbit polyclonal anti-GFP antibody (1:500, 1 h, Abcam), followed by secondary antibody Alexa Fluor 488 goat anti-rabbit (1:250, 1 h, ThermoFisher). DNA was stained with 1 µg/mL 4′,6′-diamidino-2-phenylindole (DAPI), and slides were mounted with Vectashield (Vector Laboratories). kDNA volumetric analyses were conducted as previously described (22). Images were acquired using an inverted Nikon Ti2-E wide-field fluorescence microscope with a Nikon Plan Apo λ 60x 1.45 numerical aperture objective lens. Z stacks with a step size of 0.2 µm were deconvoluted using the Landweber algorithm within NIS Elements, a closed-source analysis tool developed by Nikon Instruments.

## RESULTS

### Design and establishing a triple inducible system

In our work to expand the molecular toolbox for functional genomics in *Trypanosoma brucei*, we transitioned from using the well-established single inducible Tet-ON system (26) to a dual inducible system for robust RNAi complementation studies. Dual induction relies on the procyclic cell line SMUMA that constitutively expresses Tet and Van repressors, as well as T7 RNA polymerase (22). We identified an opportunity to provide additional flexibility by incorporating the previously reported cumate (Cym) inducible components (21, 27) that could allow for the independent regulation of three processes in a single cell line.

**Fig 1:**
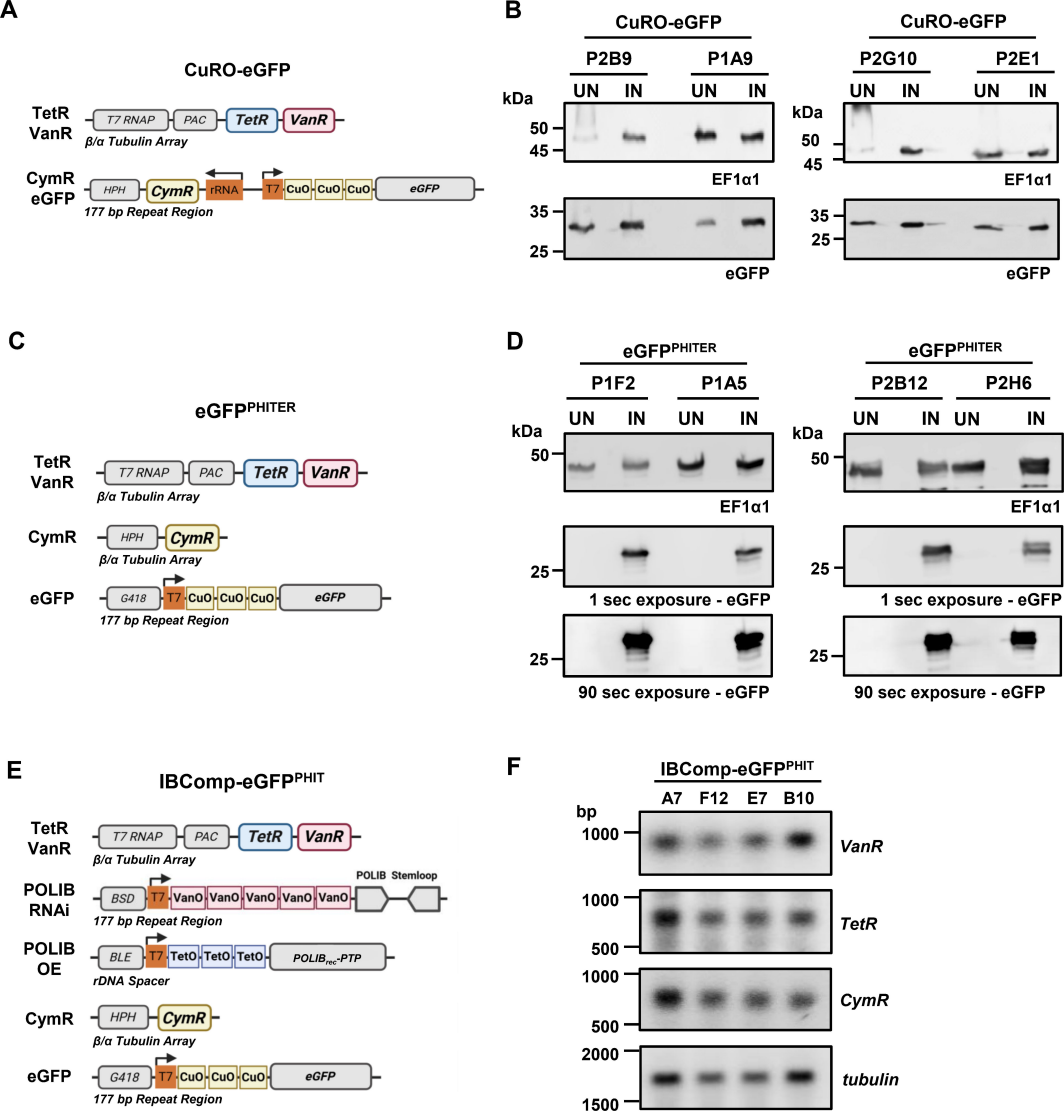
Triple inducer systems. (**A**) Diagram of plasmids integrated into the CuRO-eGFP cell line. Cumate repressor (CymR), cumate operator (CuO), neomycin phosphotransferase II (G418), puromycin-N-acetyl-transferase (PAC), tetracycline repressor (TetR), T7 RNA polymerase (T7RNAP), vanillic acid repressor (VanR). (**B**) Western blot detection of eGFP and EF1α1 loading control from CuRO-eGFP clonal cell lines. A total of 2 × 10^6^ cell equivalents were loaded per lane. (**C**) Diagram of plasmids integrated into the eGFP^PHITER^ cell line. Annotation as described in panel **A**. (**D**) Western blot detection of eGFP and loading control EF1α1 following a 48 h induction of eGFP^PHITER^ clones with 25 μg/mL Cym. A total of 2 × 10^6^ cell equivalents were loaded per lane. (**E**) Diagram of plasmids integrated into the IBComp-eGFP^PHIT^ cell line. Blasticidin S-deaminase (BSD), vanillic acid operator (VanO), tetracycline operators (TetO), bleomycin resistance gene (BLE). Other annotation as described in panel A. (**F**) Northern blot of total RNA from IBComp-eGFP^PHIT^ clonal cell lines. Membrane was probed for individual repressor mRNAs, stripped, and then reprobed. *tubulin*, loading control.

Initially, we set out to design a vector that housed both the cumate repressor (CymR) and a cumate (Cym) inducible eGFP reporter under a single drug selectable marker to integrate into the commonly used 177 bp repeat region located near the telomeric ends of the ~100 minichromosomes (Fig. 1A). We transfected pCuRO-eGFP into the dual inducible parental cell line SMUMA. Of 10 clonal cell lines, only four displayed cumate-inducible eGFP expression following 48 h of 25 µg/mL Cym induction with detectable eGFP expression in uninduced controls, indicating the system was leaky (Fig. 1B).

Our second attempt to create a triple inducible cell line relied upon separating the elements onto two vectors similar to previous work (21). The CymR gene is housed within pCymRHyg to integrate into the β/α tubulin repeat array and the expression vector, pCuO-eGFP, contains three cumate operators upstream of an eGFP reporter gene to integrate into the 177 bp repeat region (Fig. 1C). SMUMA was transfected with pCymRHyg to create a basal, triple-inducible parental cell line called PHITER (puromycin hygromycin inducible triad of exogenous repressors), and 10 clonal cell lines were generated. Several PHITER clones were subsequently transfected with pCuO-eGFP to characterize cumate-inducible expression using eGFP as a reporter. Following induction with 25 µg/mL Cym for 48 h, four eGFP^PHITER^ clones showed varying levels of eGFP protein expression, while no expression was detected in the uninduced cells (Fig. 1D). Longer exposures (90 s) showed no expression of eGFP in the uninduced controls. These results demonstrate that separating the Cym elements onto two vectors eliminated the problem of leakiness in the uninduced controls.

To demonstrate independent control by all three inducers, we transfected pCymRHyg and pCuO-eGFP into the previously characterized RNAi complementation cell line, IBComp^VaT^ (22), to create IBComp-eGFP^PHIT^, thus allowing Cym-inducible eGFP expression in addition to Van-inducible *POLIB* RNAi and Tet-inducible POLIB_rec_-PTP overexpression within a single cell line (Fig. 1E). Both the pCymRHyg and pJ1173 integration events occur within the β/α tubulin gene repeat region on chromosome 1. This region contains four tandem repeats of the tubulin genes with several intergenic regions available for integration events. Therefore, we performed northern blot analyses to confirm expression of each repressor mRNA in the four clones (Fig. 1F). IBComp-eGFP^PHIT^ clone P1F12 was selected as the cell line to analyze proof-of-concept for triple induction based on repressor expression and similar *POLIB* RNAi complementation results as previously published (22).

To determine if the triple-inducible cell line IBComp-eGFP^PHIT^ was able to express eGFP across a range of Cym concentrations (0.5–10 μg/mL) that were added to the cells for 48 h, cell pellets were collected and then analyzed by western blot analysis. No eGFP expression was observed in uninduced cells even upon a longer exposure of 5 min (Fig. S2A). Expression of eGFP was detected from 1 μg/mL Cym and consistently increased as the Cym concentration increased to 10 μg/mL (Fig. S2A). Cells induced with 5 μg/mL of Cym or greater showed faster migrating bands representing truncated protein or degradation products (Fig. S2A). We further tested 3 and 5 μg/mL Cym for sustained expression of eGFP over an 8-day induction. While some variation in the amount of eGFP was detected using 5 μg/mL, there was consistent expression of eGFP using 3 μg/mL Cym throughout the 8-day induction (Fig. S2B). We also tested whether Cym concentrations impacted the fitness of the cells. No significant fitness impact was detected by inducing the cells with a range of Cym concentrations (3, 5, 10 μg/mL) or by expression of eGFP over an 8-day induction (Fig. S2C). Based on these data, 3 μg/mL Cym was used for subsequent inductions.

### Independent gene expression in IBComp-eGFP^PHIT^ cell line

**Fig 2:**
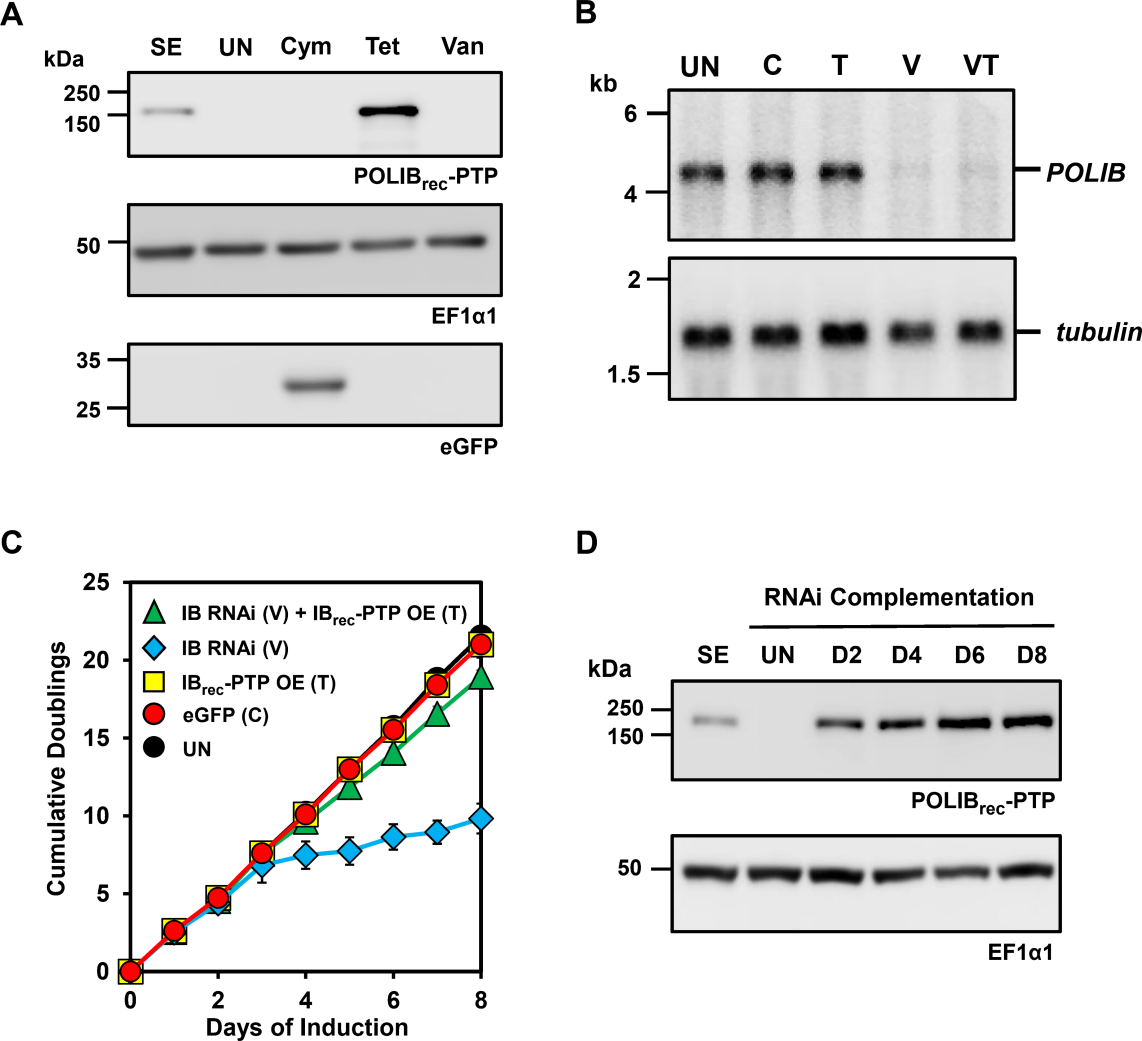
Independent gene expression in IBComp-eGFP^PHIT^ cells. (**A**) Western blot detection of POLIB_rec_-PTP, eGFP, and EF1α1 from IBComp-eGFP^PHIT^ cells independently induced with Tet (4 µg/mL), Van (250 µM), and Cym (3 µg/mL) over an 8-day induction. SE, single expressor cell line representing allelically tagged POLIB-PTP (4 × 10^6^ cell equivalents). A total of 2 × 10^6^ cell equivalents were loaded per lane. (**B**) Northern blot of total RNA from IBComp-eGFP^PHIT^ cells. U, uninduced; Cym, induced with 3 µg/mL Cym; Tet, induced with 4 µg/mL Tet; Van, induced with 250 µM Van; VaT, induced with Van and Tet; all conditions induced for 48 h. Top, probing of *POLIB* mRNA. Bottom, probing of *tubulin* mRNA as a loading control. (**C**) IBComp-eGFP^PHIT^ was grown in the absence or presence of Tet (T-4 µg/mL), Van (V-250 µM), Cym (C-3 µg/mL), and Tet and Van combined (VT). Error bars represent ± s.d. of the mean from three biological replicates. (**D**) Western blot detection of POLIB_rec_-PTP and loading control EF1α1 from IBComp-eGFP^PHIT^ grown in 250 µM Van and 4 µg/mL Tet for 8 days. SE, single expressor allelically tagged POLIB-PTP control. A total of 2 × 10^6^ cell equivalents were loaded per lane.

One advantage of the triple inducible system is that all the single-inducer controls can be performed in a single cell line. To determine whether the Van, Tet, and Cym inducible systems could operate independently of one another, each inducer was added separately to the IBComp-eGFP^PHIT^ cell line. Cells were cultured for 8 days with the addition of either 3 μg/mL Cym, 4 μg/mL Tet, or 250 μM Van. After 48 h, ectopic POLIB_rec_-PTP and eGFP expression were assessed by western blotting, and endogenous *POLIB* mRNA was quantified via northern blotting. Cells induced with Tet expressed POLIB_rec_-PTP (10.1-fold greater than allelically tagged POLIB-PTP, SE) with no detectable eGFP, while cells induced with Cym expressed eGFP with no detectable expression of POLIB_rec_-PTP (Fig. 2A).

Induction with Van did not lead to expression of either POLIB_rec_-PTP or eGFP while endogenous *POLIB* mRNA was depleted by 98% within 48 h (Fig. 2B). Throughout the 8-day induction, only the addition of Van led to a significant loss of fitness (LOF) with cell doublings reaching only 9.8 compared to 21.5 for uninduced cells, a hallmark associated with *POLIB* RNAi (16, 18, 22). Additionally, the stem-loop transcript and degradation products are only detected when RNAi is induced with Van (Fig. S3A). Cells induced with Tet or Cym reached 20.8 and 21 doublings, respectively (Fig. 2C). These data confirm that the three inducers operate independently and do not lead to promiscuous expression.

To determine if *POLIB* RNAi complementation was still functioning in the triple induction system, IBComp-eGFP^PHIT^ cells were simultaneously induced with Van and Tet for 8 days. Consistent expression of POLIB_rec_-PTP was confirmed with a sevenfold increase compared to the allelically tagged POLIB-PTP (SE) (Fig. 2D). Similar to RNAi alone, induction with Van and Tet resulted in a 99% knockdown of endogenous *POLIB* mRNA after 48 h of induction (Fig. 2B). Lastly, there was a near-complete rescue of the RNAi LOF during POLIB complementation with cells reaching 18.9 doublings by day 8, phenocopying results for the dual inducible system (22) (Fig. 2C).

### Selective and concurrent gene expression in IBComp-eGFP^PHIT^ cell line

**Fig 3:**
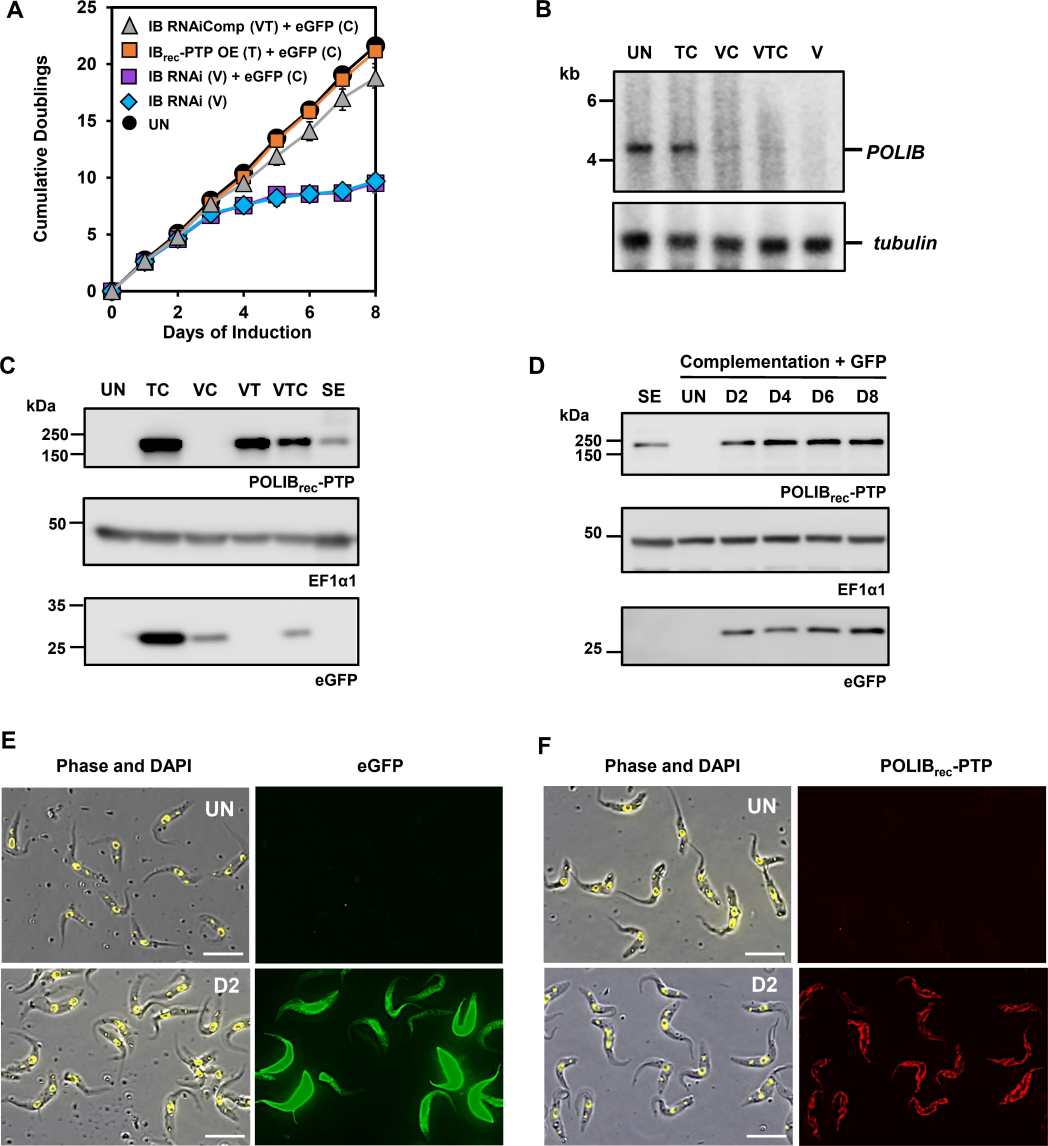
Concurrent gene expression in IBComp-eGFP^PHIT^ cells. (**A**) IBComp-eGFP^PHIT^ was grown in the absence or presence of Van (250 µM), or inducer combinations; Cym (3 µg/mL) and Tet (4 µg/mL) for 8 days. Error bars represent ± s.d. of the mean from three biological replicates. (**B**) Northern blot of total RNA from IBComp-eGFP^PHIT^ cultured for 48 h in the presence of inducer combinations. U, uninduced; TC, Tet+Cym; VC, Van+Cym; VTC, Van+Tet+Cym; V, Van. Top, probing of *POLIB* mRNA. Bottom, probing of *tubulin* mRNA as a loading control. (**C**) Western blot of POLIB_rec_-PTP, eGFP, and EF1α1 loading control from IBComp-eGFP^PHIT^ cells concurrently induced with varying combinations of Cym, Tet, and Van for 8 days. SE, single expressor allelically tagged POLIB-PTP control. A total of 2 × 10^6^ cell equivalents were loaded per lane. (**D**) Western blot of eGFP, POLIB_rec_-PTP, and EF1α1 loading control from IBComp-eGFP^PHIT^ cells concurrently induced with Cym, Tet, and Van for 8 days. SE, single expressor allelically tagged POLIB-PTP control. A total of 2 × 10^6^ cell equivalents were loaded per lane. (**E**) Representative images of eGFP expression for IBComp-eGFP^PHIT^ in the presence or absence of Cym+Tet+Van for 48 h. DAPI staining (yellow); eGFP expression (green). Size bar, 10 µm. (**F**) Representative images of POLIB_rec_-PTP IBComp-eGFP^PHIT^ in the presence or absence of Cym+Tet+Van for 48 h. DAPI staining (yellow); anti-protein A (red). Size bar, 10 µm.

To assess whether Cym induction interfered with Van and/or Tet inductions, we next evaluated how these inducers operated simultaneously in IBComp-eGFP^PHIT^ cells. The following combinations were used across 8 days of induction, Van+Cym (VC), Tet+Cym (TC), and Van+Tet+Cym (VTC), and assessed for *POLIB* mRNA knockdown, POLIB_rec_-PTP expression, and eGFP expression after 48 h. TC induction did not have a significant impact on fitness (21.2 doublings) compared to uninduced cells (21.6 doublings) and led to the highest amount of POLIB_rec_-PTP and eGFP expression with no impact on endogenous *POLIB* mRNA as expected (Fig. 3A through C). VC induction resulted in a phenocopy of RNAi alone (Van) with the characteristic LOF (9.5 doublings), a 94% reduction in *POLIB* mRNA and expression of eGFP (Fig. 3A through C). The full triple induction (VTC) also led to a 97% reduction in *POLIB* mRNA, a near-complete rescue of the RNAi LOF similar to previously reported (22) and eGFP expression (Fig. 3A through C). The stem-loop transcript and degradation products are detected when RNAi is induced with Van under all combinations (Fig. S3B). Compared to allelically tagged POLIB-PTP, TC induction led to a 12.3-fold increase in POLIB_rec_-PTP, whereas VT induction led to a slight decrease in POLIB_rec_-PTP (sevenfold) that did not significantly impact *POLIB* RNAi complementation (Fig. 2C and D and 3C). The triple induction with VTC resulted in a 6.5-fold increase above allelically tagged POLIB-PTP levels that still allowed for complementation similar to VT alone. For VC, eGFP decreased 3.2-fold compared to Cym alone with a comparable decrease of 3.6-fold for the triple VTC inductions (Fig. 3C). Although the addition of Van appeared to impact the total amount of eGFP or POLIB_rec_-PTP being produced, simultaneous induction with three independent inducers still led to a near-complete rescue of the *POLIB* RNAi LOF with 18.8 doublings and produced consistent and sustained expression of eGFP and POLIB_rec_-PTP throughout an 8-day induction (Fig. 3A and D).

Lastly, fluorescence microscopy revealed that eGFP and POLIB_rec_-PTP expression were homogenous in the cell population with eGFP expressed in the cytoplasm with some fluctuation in the total amount of protein being produced and POLIB_rec_-PTP localizing throughout the mitochondrion and concentrating near the kDNA (Fig. 3E and F). These data indicate that Cym induction did not significantly interfere with *POLIB* RNAi or overexpression of POLIB_rec_-PTP and highlight that Van induction might lead to decreased expression levels for genes under the control of Cym and Tet induction.

### Impact of vanillic acid on transgene expression

**Fig 4:**
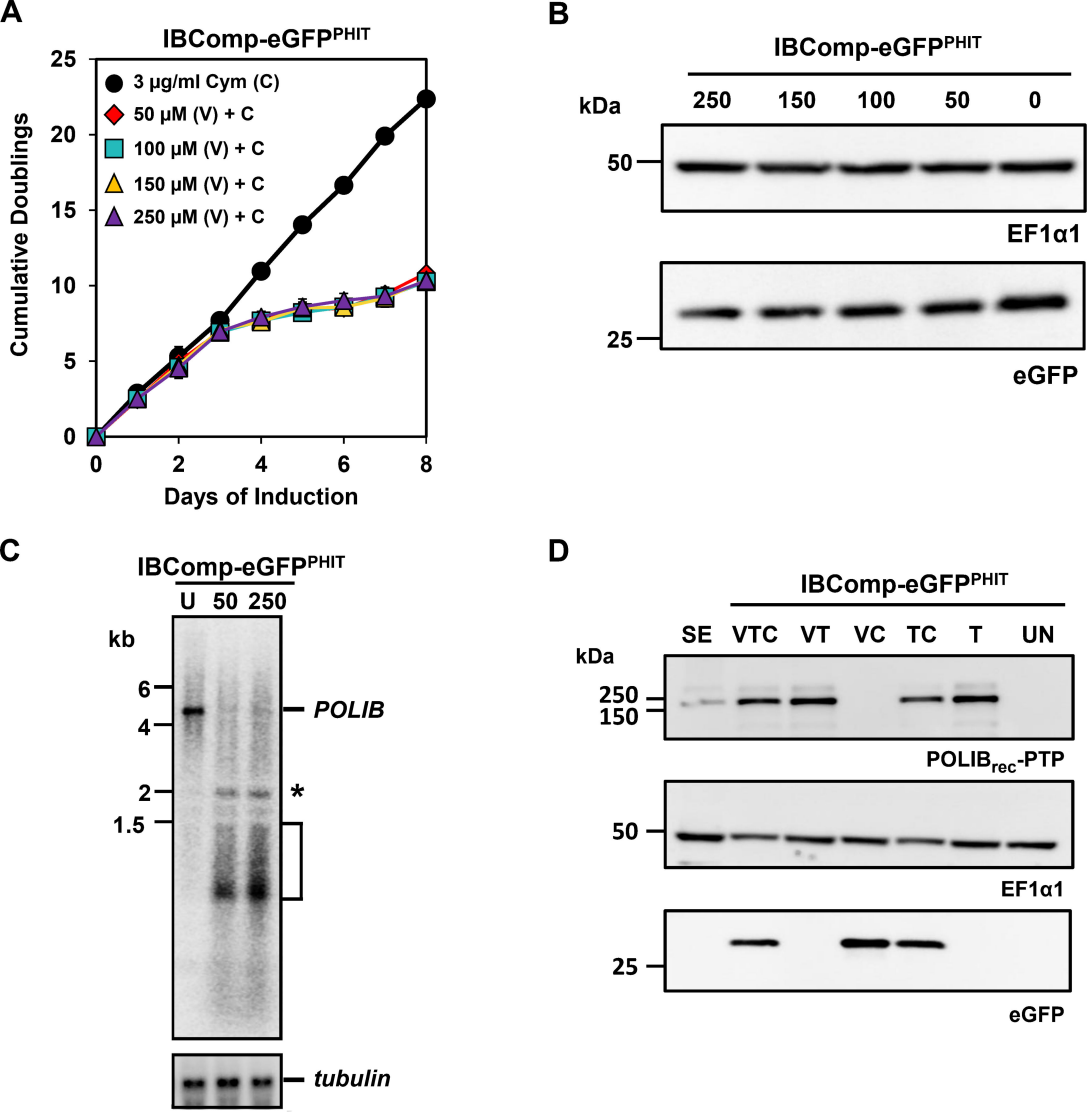
Impact of vanillic acid on transgene expression. (**A**) IBComp-eGFP^PHIT^ was grown in 3 µg/mL Cym and in the absence or presence of Van (250, 150, 100, and 50 µM) for 8 days. Error bars represent ± s.d. of the mean from three biological replicates. (**B**) Western blot of eGFP and EF1α1 loading control from IBComp-eGFP^PHIT^ cells grown in the presence of Cym and the absence or presence of varying Van concentrations for 8 days. (**C**) Northern blot of total RNA from IBComp-eGFP^PHIT^ cultured for 48 h in the presence of 50 or 250 µM Van. U, uninduced. Top, probing of *POLIB* mRNA; bottom, probing of *tubulin* mRNA as a loading control. *, POLIB stem-loop transcript induced with Van; bracket, degradation products during RNAi. (**D**) Western blot of POLIB_rec_-PTP, eGFP, and EF1α1 loading control from IBComp-eGFP^PHIT^ cells concurrently induced with Cym, Tet, and Van for 8 days. SE, single expressor allelically tagged POLIB-PTP control. A total of 2 × 10^6^ cell equivalents were loaded per lane.

Previous reports indicate variable impacts on fitness when cells are grown in the presence of 250 µM Van (20, 28). We hypothesized that the high concentration of Van could be impacting the amount of protein expressed from the transgene constructs. Previously, 250 µM Van was chosen to maximally induce *POLIB* RNAi; however, lower concentrations were never tested. To further evaluate the impact of Van in the triple inducible system, we grew IBComp-eGFP^PHIT^ cells in 3 µg/mL Cym and various concentrations of Van (250, 150, 100, and 50 µM). For all Van concentrations, LOF during *POLIB* RNAi phenocopied the original 250 µM conditions (Fig. 4A). Importantly, incremental decreases in the Van concentration led to corresponding increases in eGFP expression compared to eGFP levels in the absence of Van (Fig. 4B). While 250 µM Van decreased eGFP expression by 39%, 50 µM Van minimally impacted eGFP (16% reduction). Northern blot confirmed that *POLIB* mRNA was still efficiently reduced by 94%, and the stem-loop transcript and degradation products were minimally impacted by using a lower concentration of the RNAi inducer (Fig. 4C). We also assessed the impact of 50 µM Van on co-induction with Tet in the triple induction scenario. The lower Van concentration now showed no impact on the expression of POLIB_rec_-PTP compared to Tet induction alone, and there was a minimal decrease in eGFP expression when IBComp-eGFP^PHIT^ cells were grown with all three inducers (Fig. 4D). Note that the VTC lane had lower total protein loaded. Finally, we evaluated the impact of varying Van concentrations on the fitness of PHITER cells. This cell line expresses the three repressors but lacks the gene-specific constructs. All concentrations of Van resulted in a minimal impact on fitness, and the DMSO solvent alone had no impact on PHITER cells (Fig. S4).

### Temporal expression of eGFP during RNAi complementation

**Fig 5:**
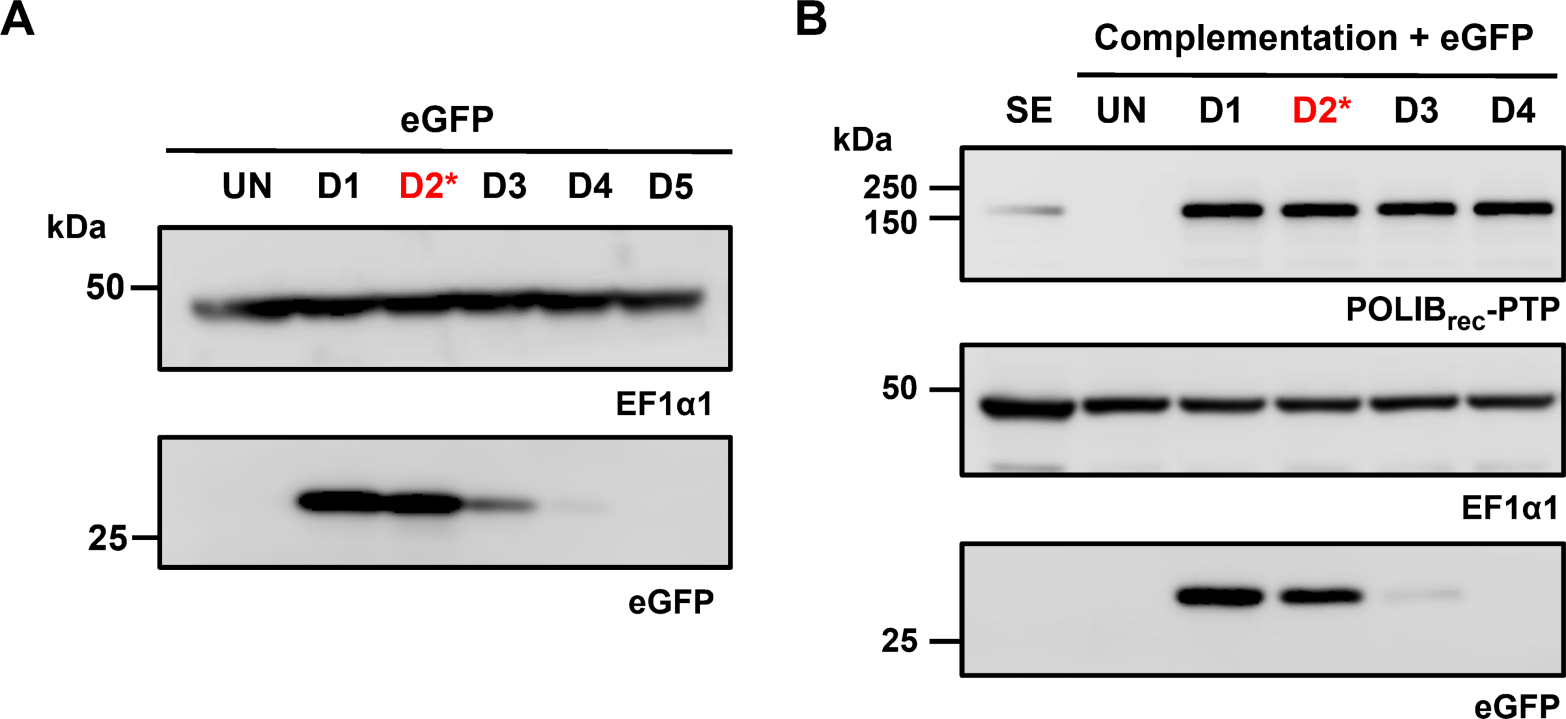
Temporal expression of eGFP in the triple inducer system. (**A**) Western blot detection of eGFP and EF1α1 loading control from IBComp-eGFP^PHIT^ cells induced with Cym (3 µg/mL), for 2 days then in the absence of Cym for subsequent days. D2*, sample taken from cell before Cym inducer was removed. A total of 2 × 10^6^ cell equivalents were loaded per lane. (**B**) Western blot detection of eGFP, POLIB, and EF1α1 loading control from IBComp-eGFP^PHIT^ cells induced with Van, Tet, and Cym for 2 days then in the absence of Cym for subsequent days. D2*, sample taken from cell before Cym inducer was removed. SE, single expressor cell line control. A total of 2 × 10^6^ cell equivalents were loaded per lane.

Another advantage of the triple inducible system would be to control when a gene of interest is expressed while performing other manipulations, such as RNAi complementation. Therefore, we tested whether eGFP expression could be controlled temporally during Cym induction and *POLIB* RNAi complementation by growing IBComp-eGFP^PHIT^ cells with inducer and then removing the Cym inducer after 48 h. Removal of Cym resulted in a decrease in eGFP expression within 24 h of removing Cym with some residual eGFP expression detected the following day (Fig. 5A). Similarly, removal of Cym during RNAi complementation resulted in more rapid reduction of eGFP expression within 24 h after removing Cym (Fig. 5B). Collectively, these data confirm the specificity of three independent inducers, ability to tune the amount of Cym and to control when Cym induction occurs. Together, the results establish proof of principle for a triple inducible system in procyclic *T. brucei* that can be used for elegant molecular genetic studies during RNAi complementation.

### Overexpression of PIF2 maxicircle helicase during *POLIB* RNAi

Previous studies determined that POLIB has an essential role in minicircle replication (18, 22), but a role in maxicircle replication was never evaluated. PIF2 is the *T. brucei* mitochondrial helicase that catalyzes the essential and rate-limiting step in maxicircle replication (10). Overexpression of PIF2 led to a sixfold increase in maxicircles with minimal impact on minicircles. If a DNA Pol is involved in maxicircle replication, then depletion of that Pol would result in suppression of maxicircle overreplication. To further test the triple inducible system and determine whether POLIB is involved in maxicircle replication, we transfected pCuO-PIF2FLAG into IBComp^PHIT^ to create IBComp-PIF2^PHIT^, thus allowing Cym inducible PIF2-FLAG overexpression in combination with Van inducible *POLIB* RNAi and Tet inducible POLIB_rec_-PTP overexpression within a single cell line.

We first independently validated the PIF2 overexpression phenotype in the triple inducible system using 25 µg/mL Cym to achieve high levels of PIF2-FLAG. Cells grown for 8 days in the presence of Cym displayed an LOF with cell doublings reaching only 10.8 compared to 22.3 in uninduced cells (Fig. S5A). PIF2-FLAG was consistently expressed while no protein was detected in uninduced cells (Fig. S5B). PIF2-FLAG was expressed homogenously across the clonal cell population, localizing throughout the single mitochondrion with signal concentrated near the kDNA (Fig. S5C). Volumetric analysis revealed an increase in the number of cells with abnormally large kDNA and cells lacking kDNA (Fig. S5D). These results are consistent with the previously reported segregation defects associated with PIF2 overexpression, including the presence of cells containing a thread-like structure connecting daughter networks that contains maxicircle DNA called the nabelschnur, and small kDNA (Fig. S5E).

**Fig 6:**
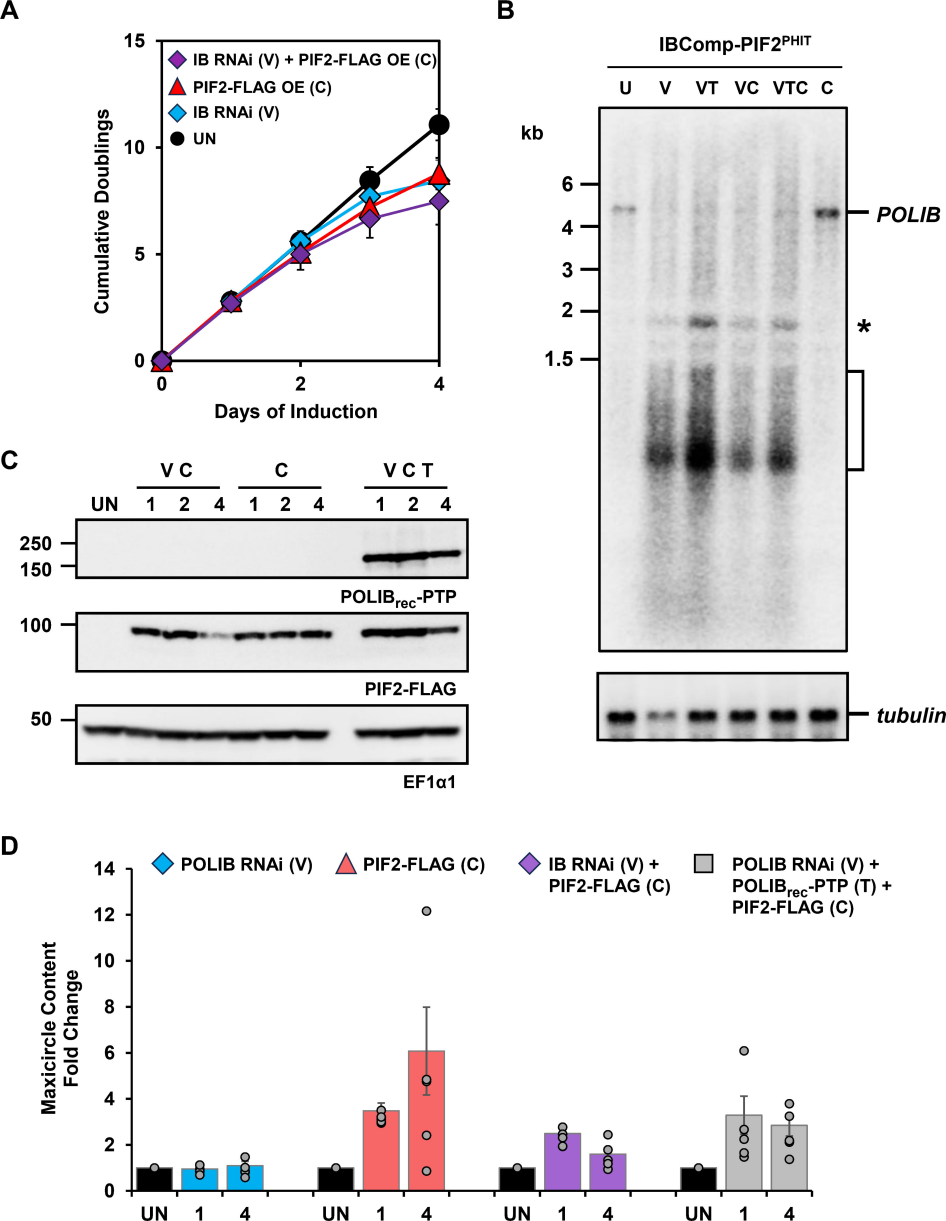
Overexpression of a maxicircle helicase during *POLIB* RNAi. (**A**) IBComp-PIF2^PHIT^ was grown in the absence or presence of inducer combinations. Van (50 µM), Cym (25 µg/mL), Van+Cym, or Van+Tet+Cym (Tet, 4 µg/mL). Error bars represent ± s.d. of the mean from three biological replicates. (**B**) Northern blot of total RNA from IBComp-PIF2^PHIT^ cultured for 48 h in the presence of inducer combinations. U, uninduced; C, 25 µg/mL Cym, V, 50 µM Van, VC, Van+Cym; VT, Van+Tet (4 µg/mL); VTC, Van+Tet+Cym; *, POLIB stem-loop transcript induced with Van; bracket, degradation products during RNAi. Top, probing of *POLIB* mRNA; bottom, probing of *tubulin* mRNA as a loading control. (**C**) Western blot of POLIB_rec_-PTP, PIF2-FLAG, and EF1α1 loading control from IBComp-PIF2^PHIT^ cells concurrently induced with Cym, Tet, and Van for 4 days. SE, single expressor allelically tagged POLIB-PTP control. A total of 2 × 10^6^ cell equivalents were loaded per lane. (**D**) Quantification of maxicircle content. Total DNA from IBComp-PIF2^PHIT^ grown as in panel A was subjected to dot blot analysis using a maxicircle-specific probe. All numerical values were normalized to the tubulin signal. Data in bar graphs represent six technical replicates from two independent experiments. Error bars represent ± s.d. of the mean.

Leveraging the triple inducible system, we evaluated POLIB’s role in maxicircle replication by growing IBComp-PIF2^PHIT^ cells for 4 days in a combination of inducers. Cells induced for both *POLIB* RNAi and PIF2-FLAG overexpression (V+C) exhibited a more severe LOF than those grown in either Van or Cym alone (Fig. 6A). Triple induction resulted in growth similar to Cym alone (data not shown). *POLIB* knockdown was confirmed by northern blot showing a decrease in *POLIB* mRNA only in inductions containing Van. Interestingly, *POLIB* mRNA appeared to increase when PIF2-FLAG was overexpressed (Cym alone) (Fig. 6B). Expression of PIF2-FLAG decreased after 4 days during induction of *POLIB* RNAi and PIF2-FLAG (V+C), while PIF2-FLAG levels were consistent across the 4 days of triple induction during *POLIB* RNAi complementation. (Fig. 6C). The decrease in PIF2-FLAG protein on day 4 correlated with the exacerbated impact on fitness (V+C). To assess the impact of maxicircle overreplication, we determined the fold change in maxicircle abundance from IBComp-PIF2^PHIT^ cells induced for *POLIB* RNAi and/or PIF2-FLAG overexpression using dot-blot Southern hybridization and a maxicircle-specific probe. PIF2-FLAG overexpression alone resulted in a sixfold increase (day 4) compared to uninduced cells, consistent with the previously published results. Notably, simultaneous *POLIB* knockdown during PIF2 overexpression suppressed the overreplication of maxicircles, producing a 2.5-fold increase on day 1 and only a 1.6-fold increase on day 4 in maxicircle content compared to uninduced cells. During *POLIB* RNAi complementation and PIF2 overexpression (V+T+C), the increased maxicircle content is partially restored (2.9, day 4). Interestingly, *POLIB* RNAi alone did not impact maxicircle content over the 4-day induction (Fig. 6D). These data indicate a role for POLIB in maxicircle overreplication.

## DISCUSSION

The use of inducible expression for reverse genetics and functional genomics screens has been critical for studying gene function in the model kinetoplastid *T. brucei*. Advances include numerous RNAi library screens and an overexpression gain-of-function screen (19, 29). However, a single inducer Tet system has prohibited more powerful multiplexed screens where two or more genetic perturbations are required (30). Examples include the design of a synthetic lethal screen and a suppressor screen. Additionally, the single Tet inducible system is not optimal for RNAi complementation studies of abundant proteins (22). This work was focused on the development of a highly tractable, triple inducer system capable of independently or simultaneously inducing the expression of three ectopic genes within one cell line. A triple inducer cell line allows for more flexibility to now perform more sophisticated mechanistic studies that could never have been achieved with the single inducer Tet system or even the recently reported dual inducer system for RNAi complementation (22). Most importantly, the triple inducer system allows for complex genetic experiments to be designed that account for temporal changes that might be necessary to address mechanistic questions.

We first attempted to engineer a parental cell line for triple induction that only required one additional drug marker, granting more flexibility in the design of downstream experiments and eliminating one transfection and selection step. However, the combined pCuRO-eGFP reporter construct only produced clones that displayed leaky eGFP expression (Fig. 1B). At this time, we are not certain why this arrangement resulted in uncontrolled eGFP expression since integration into the transcriptionally silent 177 bp repeat region is often used to prevent RNA polymerase II transcription read-through (31). In contrast, when the CymR and CuO were separated onto two vectors and introduced through two successive transfection and selection steps at separate genomic loci (177 bp repeat region and β/α tubulin array), there was robust expression of the CymR (Fig. 1F; Fig. S1B) and no evidence of leaky expression even with longer western blot exposures of up to 5 min (Fig. S2A). One concern about the successive multiple rounds of transfections and selection is the impact on the fitness of the cell lines. All of the procyclic cell lines generated in this study have doubling times (9–10 h) comparable to the parental strain SMUMA (10 h) but do grow more slowly than the Lister 427 WT line (8.5). In contrast, the widely used 29-13 cell line for the single inducer Tet system grows more slowly (~12.8 h), expresses lower levels of TetR, and displays considerable divergence in the minicircle genome (32).

To evaluate the potential of the triple inducible system we built upon the previously characterized dual inducible system for *POLIB* RNAi complementation in which RNAi is regulated by the addition of Van and expression of a recoded ectopic copy of POLIB is regulated by Tet (22). This design took into consideration previously reported relative fold increases in expression levels (Tet, 250-fold; Van, 18-fold) (20). Tet induction was used to maximize POLIB_rec_-PTP levels since this is an abundant protein, and a wide range of Van concentrations could be used for optimizing RNAi expression (50–250 µM). The previously reported cumate-inducible system was then added for regulating the expression of a third gene based on fold increase in expression and temporal control (21). We have not yet tried other combinations of the inducers. However, PHITER clonal cell lines expressing the three repressors are available to the field to use any design combination suitable for a researcher’s specific needs.

Using eGFP as a reporter in IBComp-eGFP^PHIT^ cells, we demonstrated independent control of induction with Cym, Tet, and Van with no evidence of promiscuous expression (Fig. 2A and B). Additionally, we demonstrated that Cym induction of eGFP could be tunable over a range of Cym concentrations (1–10 µg/mL) and that there was sustained expression of Cym-induced eGFP over an 8-day induction (Fig. S2A and B). Even the highest concentration of Cym does not impact the fitness of cells when tested in the parental cell line SMUMA (Fig. S2C). We also confirmed concurrent gene expression with various inducer combinations. Van+Cym still resulted in a 98% *POLIB* mRNA depletion and LOF, and Cym+Tet resulted in robust expression of both eGFP and POLIB_rec_-PTP (Fig. 3). Induction with all three inducers allowed for sustained expression of POLIB_rec_-PTP over 8 days (Fig. 3D), expression of eGFP, efficient POLIB knockdown (Fig. 3B), and a near-complete rescue of the RNAi fitness defect (Fig. 3A). These results demonstrated that all inducer combinations caused the expected expression output. Lastly, temporal control of Cym induction was demonstrated (Fig. 5), indicating that the system was capable of independent and tunable inducible gene expression to control multiple processes.

One limitation surfaced when Van induction led to decreased expression levels for genes regulated by Cym and Tet induction. Previously, 250 µM Van was reported to cause a slight fitness defect in *T. brucei* (22) and has led to dose-dependent anti-trypanosomal activity in *T. congolensi* with a reported IC50 of 768 µg/mL (28). Furthermore, synthetic vanillin derivatives and other natural phenolic compounds showed inhibitory activity against several *Leishmania* species (33, 34). Effects on *Leishmania* included mitochondrial dysfunction and altered expression of iron-dependent enzymes (Fe superoxide dismutase, ribonucleotide reductase) (33, 34). We found that all Van concentrations tested minimally impacted fitness when applied in the absence of other transgenes in the PHITER clonal cell line that expressed only the three repressors (Fig. S4A). We are not certain how Van impacts *T. brucei* biology.

Previously, the highest concentration of Van was initially selected to ensure robust knockdown of POLIB (22). However, lower Van concentrations were never tested. While lowering Van to 50 µM did not reduce POLIB knockdown efficiency or greatly impact the amount of stem-loop transcript induced (Fig. 4A and C), lowering Van concentrations did result in increasing amounts of eGFP, with 50 µM producing the highest amount of eGFP compared to Cym induction alone (Fig. 4B). When concurrent expression was tested with 50 µM Van, there was no longer a notable decrease in either eGFP or POLIB_rec_-PTP. We advise using the lowest amount of Van possible and empirically determining if there is any impact on transgene expression. Although we did not test any concentration lower than 50 µM Van, the long dsRNA constructs regularly used in *T. brucei* may be sufficient to provide ample siRNAs for efficient knockdowns.

Minicircles replicate as free molecules and are then reattached to the kDNA network for final stages of replication, including repair of nicks and gaps. In contrast, maxicircles replicate while attached to the network, making it more challenging to understand their replication dynamics. The three essential kDNA Pols represent an interesting example of paralog specialization, with POLIB displaying divergent properties compared to all other family A DNA Pols (35). To more precisely define the role of POLIB in kDNA replication, we took advantage of the kDNA network itself as an informative and sensitive reporter system for studying subtle phenotypes that might arise during replication stress. One striking example is the dramatic increase in maxicircle content during PIF2 overexpression (10) that we demonstrated by inducing the IBComp-PIF2^PHIT^ cell line with Cym only (Fig. 6D). We exploited this overt phenotype and further tested the triple inducible system by combining PIF2-FLAG overexpression with *POLIB* RNAi. Depletion of POLIB suppressed maxicircle overreplication as early as day 1 of dual induction (V+C) and continued through day 4. It is important to note that on day 4, PIF2-FLAG protein levels declined during *POLIB* RNAi. PIF2-FLAG reduction was more notable with RNAi alone compared to complementation conditions when POLIB_rec_-PTP was ectopically expressed. These data indicate a suppression of maxicircle overreplication during *POLIB* RNAi that can be partially restored when POLIB_rec_-PTP is overexpressed, demonstrating that the triple inducible system is suitable for complex mechanistic studies.

Day 4 appears to be a critical point. During *POLIB* RNAi alone, there is a significant decrease in the mean kDNA volume that is detected as early as day 2, before there is an impact on fitness, and cell cycle progression is affected shortly after (22). The HslVU protease complex acts as a negative regulator of PIF2, degrading the protein at the appropriate time in the cell cycle (10). It is possible that the combined stress of *POLIB* RNAi and PIF2 overexpression at day 4 profoundly impacted HslVU, leading to degradation of the ectopically expressed PIF2-FLAG. Alternatively, it is possible that the impact of *POLIB* RNAi on kDNA volume and cell cycle progression may force HslVU to degrade PIF2-FLAG prematurely in an attempt to maintain homeostasis.

Our study provides proof of concept for the first dynamic triple control system that allows for robust RNAi complementation and regulation of expression of a third gene. Similar to the dual induction system, the triple inducible system allows for independent and tunable gene expression to control multiple processes. Additionally, the three inducers can also be temporally modulated for simultaneous or more elegant staggered experimental designs, such as expressing proteins that specifically damage kDNA prior to performing RNAi complementation.

## Data Availability

Plasmids and corresponding sequences are available upon request, in compliance with the ASM Data Policy.

## SUPPLEMENTAL MATERIAL

Supplemental material msphere.00596-25-s0001.pdf Supplemental tables and figures.

ASM does not own the copyrights to Supplemental Material that may be linked to, or accessed through, an article. The authors have granted ASM a non-exclusive, world-wide license to publish the Supplemental Material files. Please contact the corresponding author directly for reuse.
