# Supplementary material for: A trypanosome trifecta: an independently tunable triple inducible system for genetic studies in Trypanosoma brucei
Source: mSphere. 2026 Mar 26;11(4):e00596-25. doi: 10.1128/msphere.00596-25 (PMC13123715; doi:10.1128/msphere.00596-25)
Supplement: Supplemental material — Supplemental tables and figures. [file msphere.00596-25-s0001.pdf]

Table S1: Primers used in this study.

Gene	Purpose	Primer Name	Primer Sequence (5' – 3')
Hygromycin-B phosphotransferas WVW91683.1	Gibson Cloning pSmOxNUSHyg	UM147	AGC AAT AAA GCA TCA GAA TTA TGA AAA AGC CTG AAC TCA CCG C
		UM149	CAC TTA AGC GCA GCG CCA TGC TAT TCC TTT GCC CTC GGA CGA
Neomycin phosphotransferase CAD21776	Gibson Cloning pCuO-eGFP	UM184	CGG TGT TAG GAT CTC CGA GGT CAG AAG AAC TCG TCA AGA AG
		UM185	GCC ATA AAA TAA GCT ATC ACA TGA TTG AAC AAG ATG GAT TGC ACG
Not applicable	Gibson Cloning pCuRO-eGFP	UM159	CGG TGT TAG GAT CTC CGA GGT TGT GGC CGC GCA TCC TAG G
		UM160	AAG TAG CGC TTA CGG CGT ACC GCG TTC GCG TAA GGA TCC C
Not applicable	Gibson Cloning pCymRHyg	UM192	AAA ATA GTT CAA ACG AAT TAG GCA ACC TGA ACC TTC GC
		UM193	GGA AAT GCC CCG TCC GCG TGC GCC ATG CTA TTC CTT TGC
PIF2 Tb927.11.6900	Gibson Cloning pCuRO-PIF2	UM194	TCA TCT TTA TAA TCG TTA ATC GGT GTT GCT TCC TTC GCT TG
		UM195	TCA CAC GGA TAC TCA AAG ATA TGT CTT CAA AGA CGG TAG TTT GG
POLIB Tb927.11.4690	Northern blot	MK157	CAT TCA CAG GGG TTG AAG TCA TCG C
		MK159	ACG TTC CAC CCT ACT GTA CAC TAC G
CymR O33453.1	Northern blot	UM212	GCG TGA TTG CTT AGT AAG TTG GTG
		UM213	TAC GAA TCA GTG CCC AAG TGG AGC
VanR WP_010920250.1	Northern blot	UM125	GCC AGG TCA GCG TGT GAT GAT G
		UM126	TGC TCG TAC TCT GCT GGA AGA TCC
TetR XNS37014.1	Northern blot	UM236	GCC TGG ACA AGA GCA AGG TCA TTA AC
		UM237	AAC CCG ACT CGC ACT TGA GCT GCT TC
Maxicircle	Southern blot	UM251	CTA ACA TAC CCA CAT AAG ACA G
		UM252	ACA CGA CTC AAT CAA AGC C

Table S2: Cell lines used in this study

Name	Description	Drug Selection	Reference
SMUMA (Dual Inducer)	Lister 427 T7RNAP + TetR + VanR	Puro	Armstrong et al. 2025
IBPTP/IBKO	Single Expressor POLIB-PTP	G418, Puro	Armstrong et al. 2025
$IBComp^{VaT}$	SMUMA POLIB RNAi + $POLIB_{rec}$-PTP OE	Puro, Bsd, Phleo	Armstrong et al. 2025
PHITER (Triple Inducer)	SMUMA CymR	Puro, Hyg	This Study
$eGFP^{PHITER}$	PHITER eGFP	Puro, Hyg, G418	This Study
$IBComp^{PHIT}$	$IBComp^{VaT}$ CymR	Puro, Bsd, Phleo, Hyg	This Study
IBComp-$eGFP^{PHIT}$	$IBComp^{PHIT}$ eGFP	Puro, Bsd, Phleo, Hyg, G418	This Study
IBComp-$PIF2^{PHIT}$	$IBComp^{PHIT}$ PIF2	Puro, Bsd, Phleo, Hyg, G418	This Study

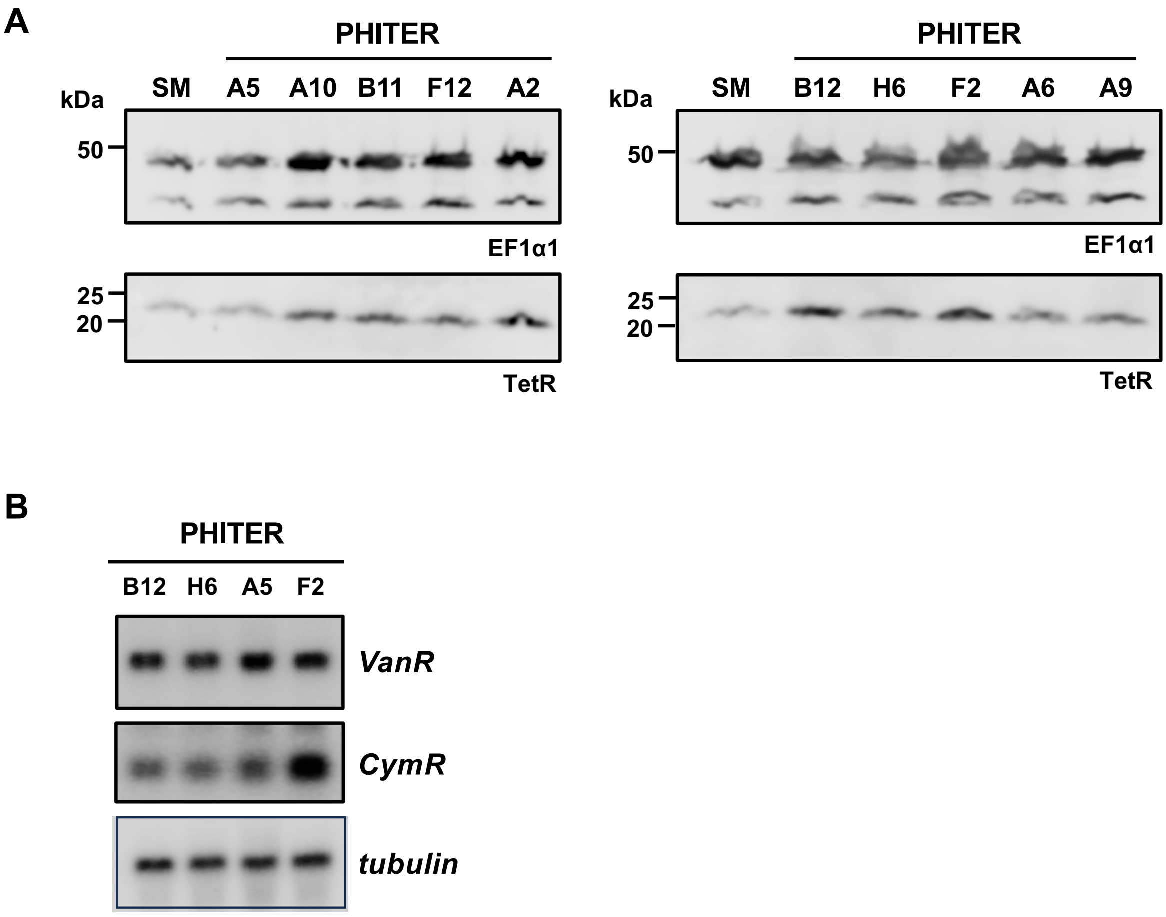

Figure S1. Characterization of PHITER clonal cell lines.

(A) Western blot detection of TetR and EF1α1 loading control from PHITER clonal cell lines. 2 x 10^6 cell equivalents were loaded per lane**. (B)** Northern blot of total RNA from PHITER clonal cell lines. Top, probing for *VanR* mRNA; Middle, probing for *CymR* mRNA; probing for tubulin (loading control). Representative blot was stripped and reprobed to detect the three transcripts.

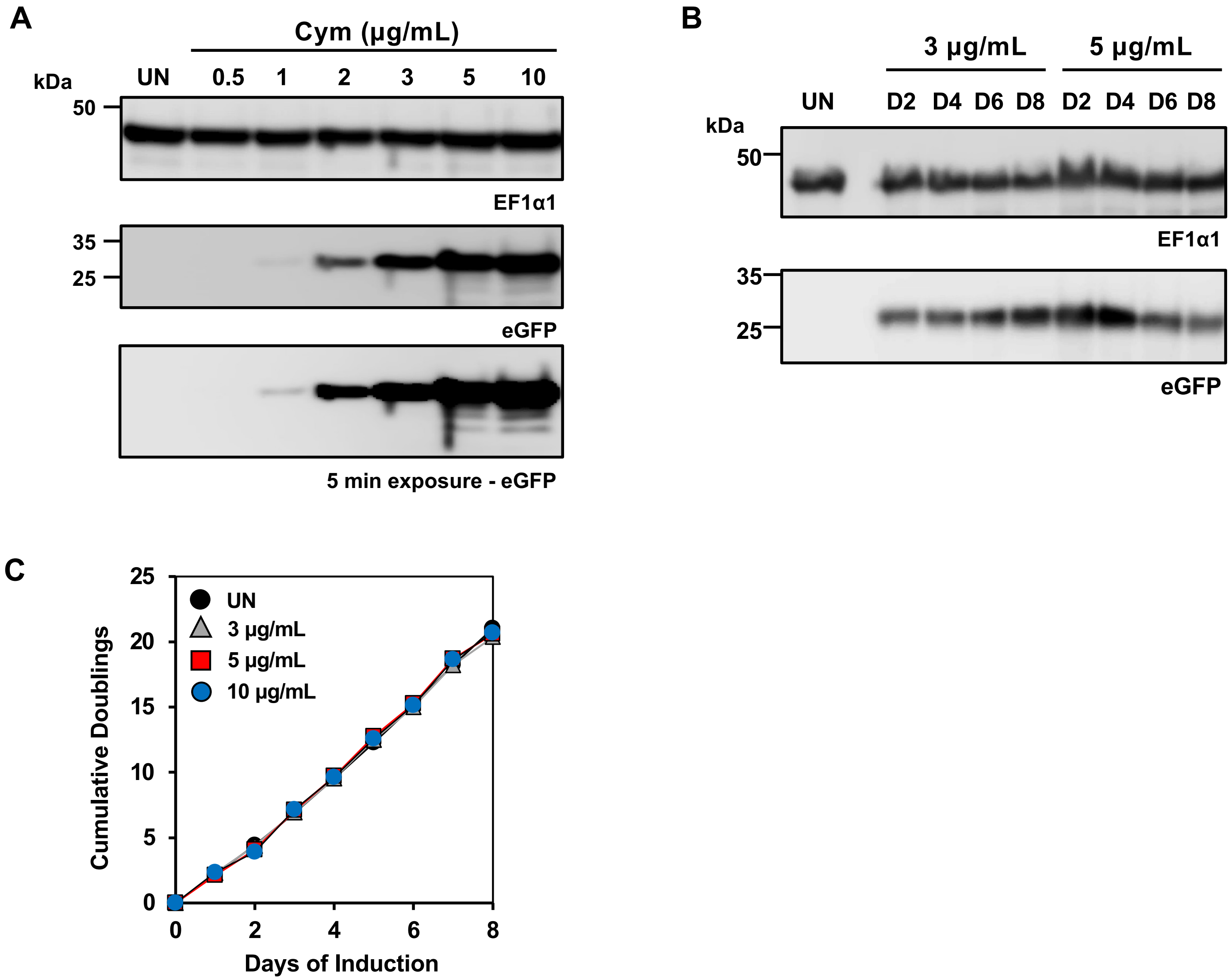

Figure S2. Cumate Inducible eGFP Expression in IBComp-eGFP^{PHIT}.

(A) Western blot detection of eGFP and EF1α1 loading control from IBComp-eGFP^{PHIT} cells induced with varying concentration of Cym for 48 hr. 2 x 10^6 cell equivalents were loaded per lane**. (B)** Western blot detection of eGFP and EF1α1 loading control from IBComp-eGFP^{PHIT} cells induced with varying concentration of Cym for 8 days. **(C)** IBComp-eGFP^{PHIT} was grown in the absence or presence of varying concentrations of Cym. Graphs represent averaged data from two biological replicates.

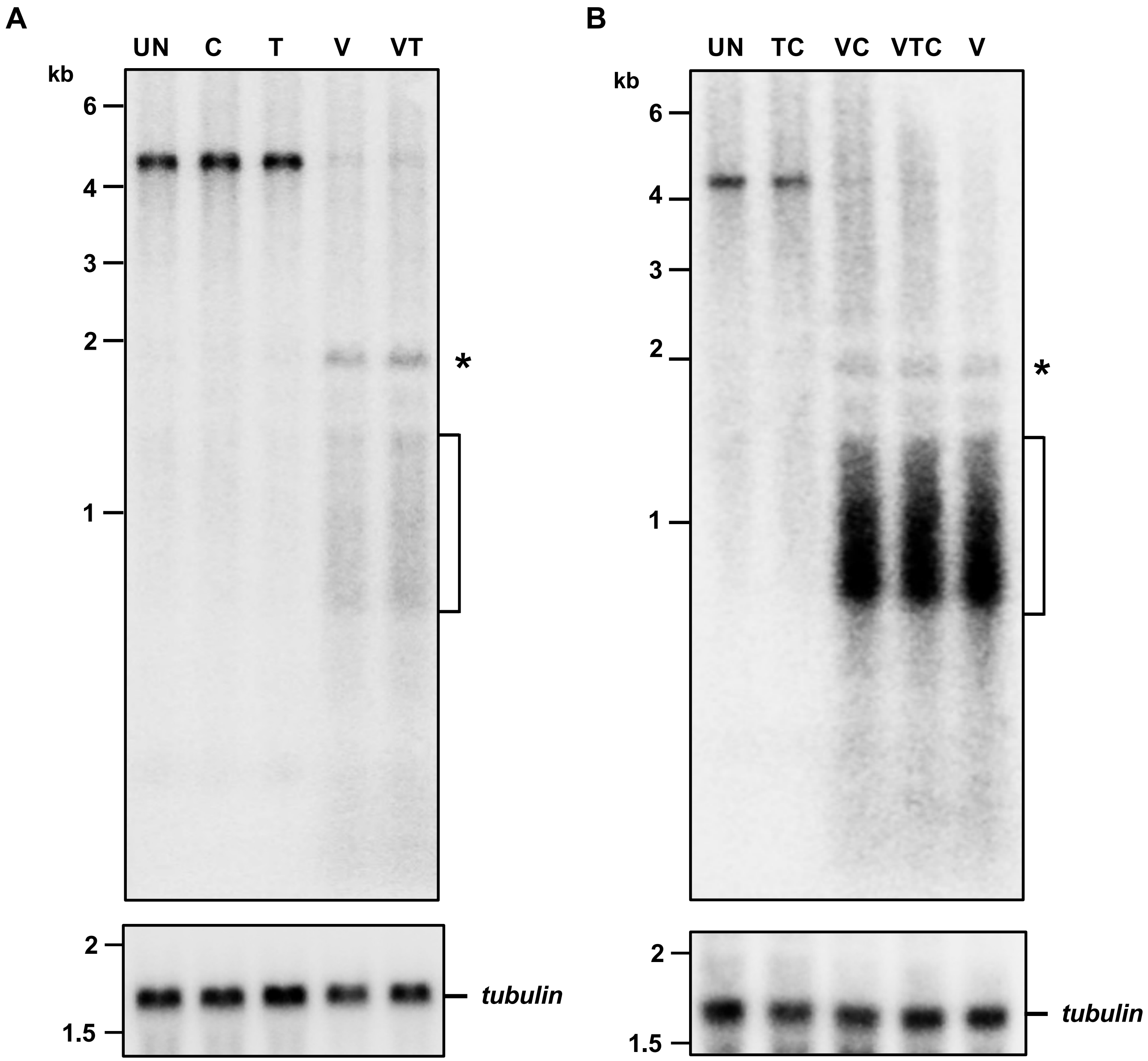

Figure S3. Confirmation of POLIB knockdown by Northern blot.

(A) Northern blot of total RNA from IBComp-eGFP^{PHIT} cells**.** This is the full gel of the same image as in Figure 2B. *, POLIB stemloop transcript induced with Van; bracket, degradation products during RNAi. **(B)** Northern blot of total RNA from IBComp-eGFP^{PHIT} cells**.** This is the full gel of the same image as in Figure 3B. *, POLIB stemloop transcript induced with Van; bracket, degradation products during RNAi.

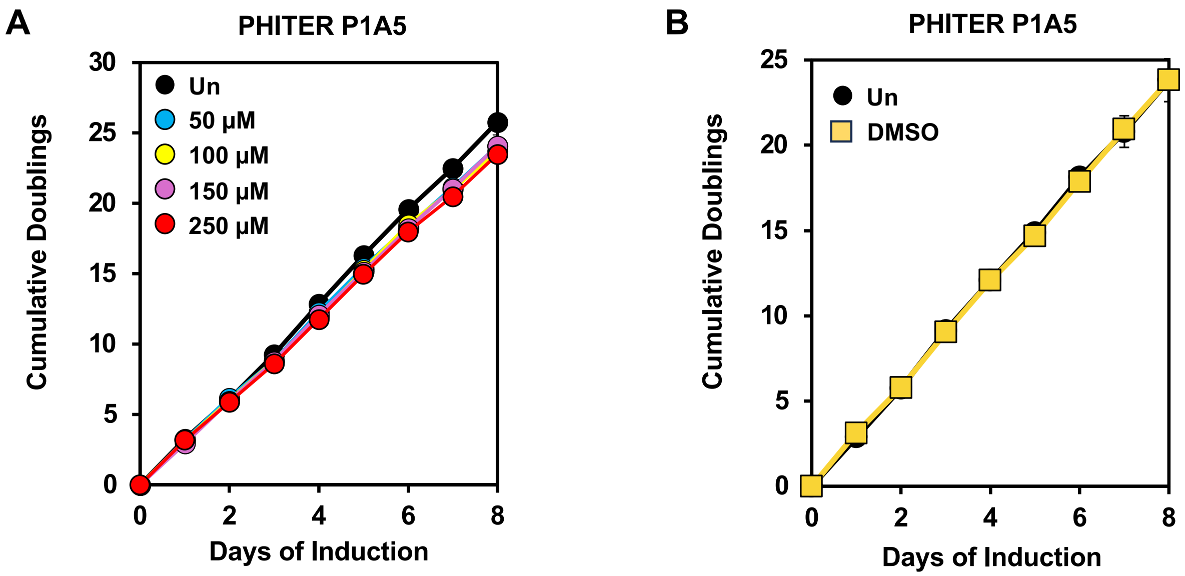

Figure S4. Effect of DMSO and Varying Van concentrations on PHITER P1A5 fitness

(A) PHITER clone P1A5 was grown in the absence or presence of varying concentration of Van. Graph represents the averaged data from three biological replicates. Error bars are too small to be seen. **(B)** PHITER clone P1A5 was grown in the absence or presence of DMSO. Graph represents averaged data from two biological replicates.

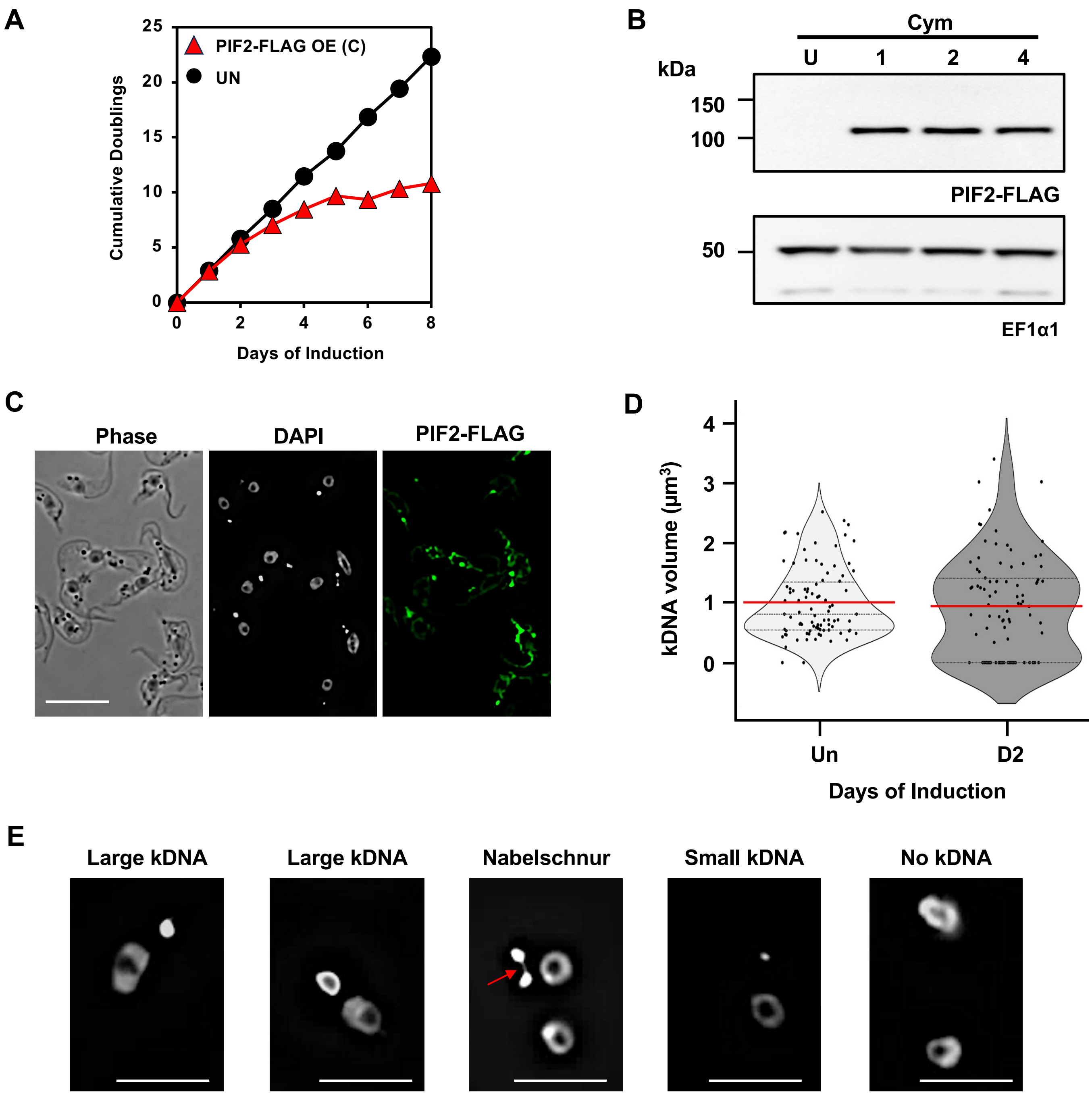

Figure S5. Cumate Inducible Maxicircle Helicase PIF2 overexpression.

(A) IBComp-PIF2^{PHIT} was grown in the absence or presence of 25 µg/ml Cym. **(B)** Western blot detection of PIF2-FLAG and EF1α1 loading control from IBComp-PIF2^{PHIT} cells induced with 25 µg/ml Cym for 4 days. **(C)** Representative images of PIF2-FLAG expression for IBComp-PIF2^{PHIT} in the presence or absence of 25 µg/ml Cym for 48 hours. Size bar, 10 mm. **(D)** Volumetric Analisis. Red line represent the mean. **(E)** Representative images of key PIF2 overexpression phenotypes.
